# Orexin receptor antagonists reverse aberrant dopamine neuron activity and related behaviors in a rodent model of stress-induced psychosis

**DOI:** 10.1038/s41398-021-01235-8

**Published:** 2021-02-08

**Authors:** Hannah B. Reiley, Stephanie M. Perez, Jennifer J. Donegan, Daniel J. Lodge

**Affiliations:** 1 Department of Pharmacology and Center for Biomedical Neuroscience, University of Texas Health Science Center, San Antonio, TX 78229 USA; 2 South Texas Veterans Health Care System, Audie L. Murphy Division, San Antonio, USA

**Keywords:** Neuroscience, Psychology, Pharmacology, Psychiatric disorders

## Abstract

Post-traumatic stress disorder (PTSD) is a prevalent condition affecting approximately 8% of the United States population and 20% of United States combat veterans. In addition to core symptoms of the disorder, up to 64% of individuals diagnosed with PTSD experience comorbid psychosis. Previous research has demonstrated a positive correlation between symptoms of psychosis and increases in dopamine transmission. We have recently demonstrated projections from the paraventricular nucleus of the thalamus (PVT) to the nucleus accumbens (NAc) can regulate dopamine neuron activity in the ventral tegmental area (VTA). Specifically, inactivation of the PVT leads to a reversal of aberrant dopamine system function and psychosis-like behavior. The PVT receives dense innervation from orexin containing neurons, therefore, targeting orexin receptors may be a novel approach to restore dopamine neuron activity and alleviate PTSD-associated psychosis. In this study, we induced stress-related pathophysiology in male Sprague Dawley rats using an inescapable foot-shock procedure. We observed a significant increase in VTA dopamine neuron population activity, deficits in sensorimotor gating, and hyperresponsivity to psychomotor stimulants. Administration of selective orexin 1 receptor (OX_1_R) and orexin 2 receptor (OX_2_R) antagonists (SB334867 and EMPA, respectively) or the FDA-approved, dual-orexin receptor antagonist, Suvorexant, were found to reverse stress-induced increases in dopamine neuron population activity. However, only Suvorexant and SB334867 were able to reverse deficits in behavioral corelates of psychosis. These results suggest that the orexin system may be a novel pharmacological target for the treatment of comorbid psychosis related to PTSD.

## Introduction

Post-traumatic stress disorder (PTSD) is a psychiatric disorder that develops following exposure to a traumatic event and is characterized by flashbacks to the traumatic event, avoidance of event-related stimuli, hypervigilance, and cognitive deficits^1^. In addition, 80–90% of individuals diagnosed with PTSD receive a comorbid psychiatric diagnosis, including depression, anxiety, and psychosis^2–5^. While PTSD and comorbid depression and anxiety have been extensively studied, less is known about psychosis in PTSD patients, despite the fact that up to 64% of individuals with PTSD are diagnosed with comorbid psychosis^5,6^. Antipsychotic medications are commonly prescribed and effectively lessen symptoms of psychosis, however, they produce many unwanted side effects that often result in poor patient compliance^7^. Although it has been repeatedly demonstrated that hyperactivity in the dopamine system contributes to symptoms of psychosis, targeting the dopamine system directly results in unwanted side effects. Interestingly, no obvious histopathology has been identified within the mesolimbic dopamine neurons of patients with psychosis^8,9^. Rather, the pathology appears to lie in upstream brain regions that regulate dopamine neuron activity^10,11^. One such region of interest, known to regulate dopamine system function, is the paraventricular nucleus of the thalamus (PVT). We have previously demonstrated that chemogenetic activation of neurons projecting from the PVT to the nucleus accumbens (NAc) produces a significant increase in ventral tegmental area (VTA) dopamine neuron population activity, defined as the number of neurons firing spontaneously^11^. Conversely, inhibition of this pathway, in a rodent model used to study the physiological and behavioral correlates of psychosis, can reverse aberrant VTA dopamine neuron activity^12^. Additionally, the PVT has been shown to be hyperactive following stress, suggesting increased signaling from the PVT to the NAc, following stress, may contribute to symptoms of psychosis in individuals with PTSD^13^.

Orexin-containing neurons, originate in the lateral hypothalamus and densely innervate the PVT, making them an ideal target to modulate the activity of the PVT^14,15^. The orexin system consists of two neuropeptides cleaved from a single precursor protein, orexin peptide A (OXA) and orexin peptide B (OXB) which bind to and activate two distinct Gq-protein-coupled receptors, orexin 1 receptor (OX_1_R) and orexin 2 receptor (OX_2_R). OX_1_R binds OXA with high affinity and binds OXB with much lower affinity while OX_2_R has been shown to have equal affinity for both OXA and OXB^16^. Further, both orexin receptors are highly expressed within the PVT^17,18^. The orexin system was first discovered as a modulator of sleep and appetite, but recent studies suggest this neuropeptide system plays a pleiotropic role, regulating a variety of biological processes, such as pain, cardiovascular function, and neuroendocrine regulation^19–22^. Although the orexin system is widespread and diverse in function, recent studies have shown that targeting this system may be beneficial in treating substance use disorder as well as psychosis^22–25^. Importantly, both OXA and OXB have been shown to dose-dependently increase the firing rate of PVT neurons and increase dopamine neuron population activity, without affecting the firing rate of these neurons in vivo^26,27^.

We have recent data demonstrating the ability of the dual-orexin receptor antagonist (DORA), TCS1102, to reverse aberrant dopamine neuron population activity in a rodent model used to examine psychosis-like behaviors, when administered both systemically and intracranially into the PVT^27^. However, little is known about the ability of the FDA-approved DORA, Suvorexant, to reverse psychosis-like deficits in a rodent model displaying stress-induced pathophysiology relevant to PTSD^28–30^. Here, we used a two-day inescapable foot-shock paradigm to induce robust increases in dopamine neuron population activity within the VTA. Additionally, we observed deficits in two dopamine-dependent behavioral correlates of psychosis, pre-pulse inhibition of startle (PPI) and hyperresponsivity to psychomotor stimulants (MK-801)^31–33^. We then investigated the ability of pharmacological inhibition of orexin receptor activity to reverse both increases in VTA dopamine neuron activity and deficits in dopamine-dependent behaviors. We found that of the FDA-approved DORA, Suvorexant, was able to reverse aberrant dopamine transmission in the VTA following two-day inescapable foot-shock stress. Additionally, Suvorexant attenuated stress-induced behavioral deficits in PPI and MK-801 induced locomotor activity. Furthermore, we found that systemic administration of either the selective OX_1_R antagonist, SB334867 or the selective OX_2_R antagonist, EMPA, was able to reverse increased VTA dopamine neuron activity following stress. However, deficits in PPI were only reversed following administration of SB334867, not EMPA. These findings suggest that targeting the orexin system, specifically the OX_1_R, can reverse aberrant dopamine neuron activity that contributes to psychosis-like behavior and that Suvorexant, or an OX_1_R antagonist, may be a novel therapeutic intervention for the treatment of comorbid psychosis in PTSD.

## Materials and methods

All experiments were performed in accordance with the guidelines outlined in the USPH Guide for the Care and Use of Laboratory Animals and were approved by the Institutional Animal Care and the Use Committees of UT Health San Antonio and U.S. Department of Veterans Affairs.

### Animals

Adult male Sprague Dawley rats (250–275 g) were obtained from Envigo RMS Inc. (Indianapolis, IN, USA) and used for all experiments. Rats were maintained in a temperature-controlled environment, on a 12 h/12 h light/dark cycle, with ad libitum access to food and water. Animals were randomly allocated to various experimental groups. Experimenters were blinded to treatment groups for all behavioral tests.

### Drug administration

Suvorexant, the highly selective dual-orexin antagonist, (10 mg/kg or 30 mg/kg; OX1R, Ki = 0.54 nM; OX2R, Ki = 0.57 nM^34^) or vehicle (DMSO) were administered intraperitoneally 30 min prior to electrophysiological recordings or behavioral assays. For experiments examining selective orexin receptor activity, the selective OX_1_R antagonist, SB334867 (10 mg/kg; OX_1_R, Ki = 99 nM; OX_2_R Ki = >10,000 nM^35^) the selective OX_2_R antagonist, EMPA, (10 mg/kg; OX_1_R, Ki = 900 nM; OX_2_R Ki = 1.45 nM^36^), or vehicle (10% DMSO and 10% 2-hydroxypropyl-b-cyclodextrin in sterile water) were given 20 min prior to electrophysiological recordings or behavioral assays.

### Two-day inescapable foot-shock

Rats were placed in a 30.5 × 25.4 × 30.5 cm^3^ square conditioning chamber with metal walls and a stainless-steel grid shock floor (Coulbourn Instruments, Whitehall, PA, USA). Rats randomly assigned to the stress group received a two-day inescapable shock treatment, where each day they received 60 × 15 s 0.8 mA foot shocks with an inter-trial interval (ITI) of 30 s with a 25% deviation (+/− 7.5 s). Control rats were handled daily but not exposed to conditioning chambers. Electrophysiological experiments and PPI assays were conducted 24 h following inescapable stress. MK-801 induced locomotor response assays were conducted 48 h following inescapable stress.

### In vivo extracellular dopamine neuron recordings

Rats were anesthetized with 8% chloral hydrate (400 mg/kg, i.p.) and placed in a stereotaxic apparatus. Chloral hydrate was used for all dopamine recordings to avoid significantly depressing dopamine neuron activity^37^. Supplemental anesthesia was administered to maintain suppression of limb compression withdrawal reflex. Core body temperature of 37 °C was sustained using a thermostatically controlled heating pad (PhysioSuite, Kent Scientific Coorporation, Torrington, CT, USA). Extracellular glass microelectrodes (impedance ~6–10 MΩ) were lowered into the VTA (posterior 5.3 to 5.7, lateral 0.6 to 1.0 from bregma, and −6.5 to −9.0 mm ventral of the brain surface) using a hydraulic micro-positioner (Model 640, Kopf Instruments). Spontaneously active dopamine neurons were recorded using previously established electrophysiological criteria^38,39^. Open filter settings (low-frequency cutoff: 30 Hz; high-frequency cutoff: 30 kHz) were used to identify and record dopamine neurons in the VTA. Various regions of the VTA were sampled by making multiple 6–9 vertical passes, separated by 200 µm, in a predetermined pattern. Three parameters of dopamine activity were measured and analyzed: the number of dopamine neurons firing spontaneously (population activity)^11^, basal firing rate, and proportion of action potentials occurring in bursts. Electrophysiological analysis of dopamine neuron activity was performed using commercially available computer software (LabChart version 8; ADInstruments, Colorado Springs, CO, USA) and analyzed with Prism software (GraphPad Software, San Diego, CA, USA). All orexin antagonists were administered thirty minutes prior to extracellular recordings and single-cell extracellular recordings lasted no longer than two hours post injection.

### Pre-pulse inhibition of startle response (PPI)

Rats were placed in a sound attenuated chamber (SD Instruments, San Diego, CA, USA) and allowed to acclimate to 65 dB background noise for 5 min. Rats were then exposed to ten startle-only trials (40 ms, 120 dB, 15 s average inter-trial intervals (ITI)). Next, rats were exposed to 24 trials where a pre-pulse (20 ms at 69 dB, 73 dB and 81 dB) was presented 100 ms before the startle pulse. Each pre-pulse + startle pulse and startle pulse were presented 6 times in a pseudo-random order (15 s average ITI). The startle response was measured from 10–80 ms after the onset of the startle only pulse and recorded using SR-LAB Analysis Software (SD Instruments). All orexin antagonists were administered thirty minutes prior behavioral assays.

### MK-801 induced locomotor response

Rats were placed in an open field arena (Med Associates, VT, USA) and spontaneous locomotor activity in the *x*–*y* plane was determined for 30 min by beam breaks and recorded with Open Field Activity software (Med Associates). Following a 30-min baseline recording, all rats were injected with MK-801 (75 μg/kg, i.p.). Locomotor activity was recorded for an additional 30 min immediately following dose.

### Histology

Immediately following all electrophysiological recordings, rats were rapidly decapitated. Brains were post-fixed for at least 24 h (4% formaldehyde in saline) and cryoprotected (25% w/v sucrose in PBS) until saturated. Coronal sections (25 μm) were collected on a cryostat (Leica) and mounted onto gelatin-chrom-coated slides and stained with neutral red (0.1%) and thionin acetate (0.01%) for histological verification of electrode tracks within the VTA^40^.

### Statistical analysis

Electrophysiological data were analyzed by two-way ANOVA (stress × drug). Holm–Sidak was used for all post hoc analysis, with significance determined at p < 0.05. PPI and MK-801 induced locomotor activity were analyzed using SigmaPlot (Systat Software Inc., Chicago, IL, USA). PPI Data were analyzed by three-way ANOVA and the Holm–Sidak post hoc test, with significance determined at *p* < 0.05. Locomotor data were analyzed by two separate three-way ANOVA’s (stress × drug × time), one for each of the relevant treatments (baseline, MK-801) followed by a Holm–Sidak post hoc test. All data are represented as the mean ± SEM, unless otherwise stated, with *n* values representing the number of rats per group unless otherwise specified. All data sets were checked for normality and variance was found to be similar between experimental groups. Sample sizes were determined based on our previous studies^41,42^.

### Materials

Suvorexant (Item No. 9002140; Cayman Chemical, Ann Arbor, MI, USA) was made fresh daily and dissolved in dimethylsulfoxide (DMSO). SB334867 and EMPA (Cat. No 1960 and 4558; Tocris, Minneapolis, MN, USA) were made fresh daily and dissolved in 10% DMSO and 10% 2-hydroxypropyl-b-cyclodextrin (Item No. 16169; Cayman Chemical, Ann Arbor, MI, USA) in sterile water. Chloral hydrate and DMSO were purchased from Sigma-Aldrich (St. Louis, MO, USA). All other chemicals and reagents were of either analytical or laboratory grade and purchased from standard suppliers.

## Results

### Suvorexant, a dual-orexin antagonist, reverses aberrant VTA dopamine neuron population activity

**Fig. 1:**
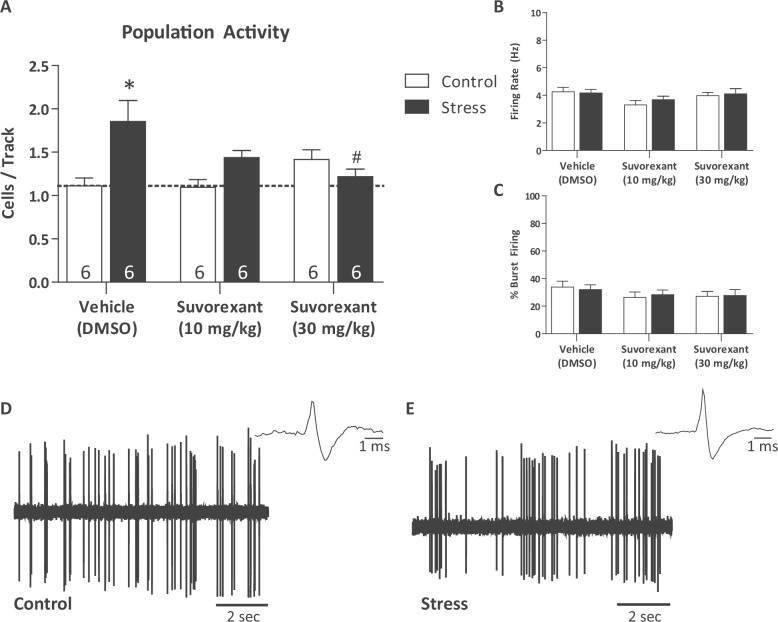
Suvorexant reverses increased dopamine neuron population activity observed after two-day inescapable foot-shock. Three parameters of dopamine neuron activity were measured: **a** population activity (average number of spontaneously active dopamine neurons per electrode track), **b** average firing rate, and **c** average percentage of spikes firing in a burst. Two-day inescapable stress increases dopamine neuron population activity which is reversed following administration of Suvorexant (**a**): *p < 0.05 compared with control/vehicle; ^#^*p* < 0.05 compared with stress/vehicle. There were no significant changes in firing rate (**b**) or bursting pattern (**c**). Representative traces from control animals (**d**) and two-day inescapable shock animals (**e**). *n* = 6 rats per group.

The dopamine hypothesis, one of the longest-standing hypotheses of psychosis, suggests an increase in dopamine activity correlates with severity of psychotic symptoms^8,9^. In rats, changes in mesolimbic dopamine activity can be examined using in vivo extracellular electrophysiology to quantify the number of spontaneously active VTA dopamine neurons^10,38,39^. Following two-day inescapable shock, we found a main effect of stress on VTA dopamine neuron population activity (Fig. 1; two-way ANOVA; factors: stress × drug; *F*_(1,35)_ = 7.731; *t* = 2.781; *P* = 0.009, *n* = 6/group). Stressed animals treated with vehicle displayed significantly higher number of spontaneously active dopaminergic cells per track when compared to control, vehicle treated animals (Holm–Sidak; *t* = 4.033, *p* = <0.001, *n* = 6/group; control/vehicle: *n* = 6 rats; 1.11 ± 0.09 cells per track; stress/vehicle: *n* = 6 rats; 1.86 ± 0.24 cells per track). This stress-induced increase in dopamine neuron activity was not observed in rats treated with either 10 or 30 mg/kg of Suvorexant and was completely reversed by the 30 mg/kg dose (*p* = 0.005, *n* = 6 rats; 1.22 ± 0.09 cells per track). No significant differences were observed in the firing rate (Stress/vehicle: *n* = 61 neurons; 4.17 ± 0.25 Hz; stress/10 mg: *n* = 49 neurons; 3.69 ± 0.25 Hz; control/30 mg: *n* = 51 neurons; 3.97 ± 0.25 Hz; stress/30 mg: *n* = 41 neurons; 4.11 ± 0.38 Hz) or percentage bursting in any of the groups (control/vehicle: *n* = 40 neurons; 33.76 ± 4.35%; stress/vehicle: *n* = 61 neurons; 32.01 ± 3.48%; control/10 mg: *n* = 37 neurons; 26.26 ± 4.03%; stress/10 mg: *n* = 49 neurons; 28.33 ± 3.41%; control/30 mg: *n* = 51 neurons; 27.16 ± 3.49%; stress/30 mg: *n* = 41 neurons; 27.69 ± 4.36%).

### Suvorexant reverses stress-induced deficits in PPI

**Fig. 2:**
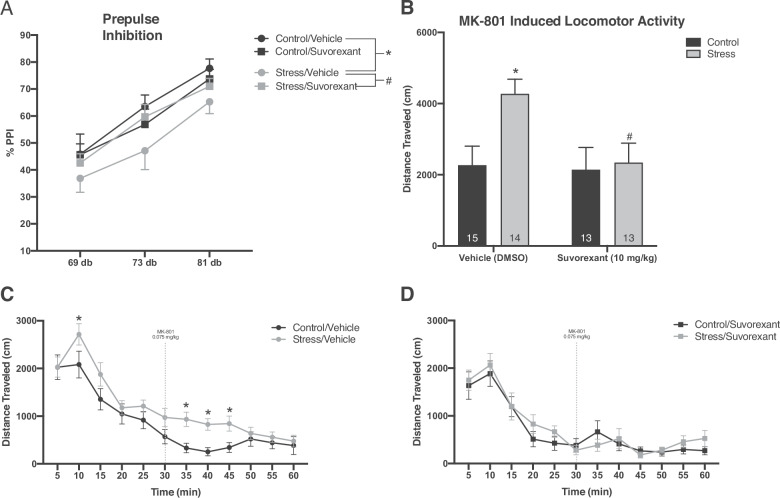
Suvorexant alleviates behavioral deficits induced after two-day inescapable foot-shock. **a** Inescapable foot-shock produced deficits in sensorimotor gating in vehicle-treated rats. *p < 0.05 compared with control/vehicle. This deficit was reversed in rats that underwent two-day inescapable shock but were treated with Suvorexant. ^#^*p* < 0.05 com*p*ared with stress/vehicle. *n* = 16 rats per group. Additionally, inescapable foot-shock induces hyperlocomotion following MK-801 administration which is reversed following administration of Suvorexant (**b**–**d**). **b** Total distance traveled after MK-801 administration was significantly higher in rats that received inescapable foot-shock. *p < 0.05 compared with control/vehicle. Hyperlocomotion was reversed in stressed animals treated with Suvorexant. #*p* < 0.05 compared with stress/vehicle. **c** Distance traveled before and after MK-801 administration in vehicle-treated rats. Stressed rats displayed sustained hyperlocomotion after MK-801. *p < 0.05 compared to control/vehicle. **d** Rats treated with Suvorexant showed no differences in baseline activity or activity following MK-801. *n* = 13–15 rats per group.

Individuals with psychosis typically display deficits in sensorimotor gating, which can be measured in both humans and animal models of the disease using pre-pulse inhibition (PPI)^31^. Initial pilot studies demonstrated that the high dose of Suvorexant (30 mg/kg) dramatically reduced spontaneous locomotor activity (by >80%) whereas the lower dose of 10 mg/kg did not (Fig. 2). For this reason, 10 mg/kg was used for the following behavioral studies. Consistent with previous literature, we found a main effect of pre-pulse intensity, demonstrated by an attenuated startle response as the pre-pulse magnitude increases (Fig. 2; *P* < 0.001; *n* = 16 rats/group). Additionally, a main effect of stress was observed (three-way ANOVA; factors: stress x drug x pre-pulse intensity; *F*_(1,191)_ = 5.845; *t* = 2.418; *P* = 0.017) demonstrating stress-induced deficits in PPI at all pre-pulse intensities. Stress/vehicle rats displayed significant deficits in PPI (Fig. 2; Holm–Sidak: *t* = 3.167, *p* = 0.002) when compared to control/vehicle. Importantly, PPI deficits observed in stress/vehicle animals were significantly reversed by Suvorexant (10 mg/kg) (Holm–Sidak: *t* = 2.014, *p* = 0.046). No differences were observed between vehicle and Suvorexant (10 mg/kg) in unstressed controls (Holm–Sidak: *t* = 0.902, *p* = 0.368). The average startle response of Suvorexant treated animals was not significantly different from control (two-way ANOVA; factors: stress × drug; *F*_(1,63)_ = 0.593; *t* = 0.770; *P* = 0.444). These results suggest repeated foot-shock stress can induce deficits in sensorimotor gating, as measured by PPI, and that these deficits can be alleviated by treatment with Suvorexant.

### MK-801 induced locomotor activity increases after stress but is reversed following administration of Suvorexant

Patients with psychosis display hyper-sensitivity to psychomotor stimulants which can be modeled in rats by measuring locomotor activity before and after administration of the NMDA antagonist, MK-801^10,41,43^. Consistent with previous literature^44^, stress/vehicle rats showed an increase in locomotor activity at baseline, compared to control/vehicle rats (Fig. 2; two-way ANOVA; factors: stress × time; *F*_(1,173)_ = 7.433; *t* = 2.726; *P* = 0.007, control/vehicle: *n* = 15 rats; stress/vehicle: *n* = 14 rats). This hyperlocomotion was exacerbated in stress/vehicle animals following MK-801 administration (*t* = 4.399; *P* < 0.001). In contrast, there were no differences in locomotor activity at baseline or after MK-801 administration between stressed and control animals treated with Suvorexant (Fig. 2). When comparing total distance traveled after MK-801 administration, across groups, we observed a main effect of stress on locomotor activity (Fig. 2; two-way ANOVA; factors: stress × drug; *F*_(1,54)_ = 4.316; *t* = 2.077; *P* = 0.043, *n* = 13–15 rats/group). Specifically, stress/vehicle animals exhibited significantly increased locomotor activity, compared to control/vehicle animals (Holm–Sidak: *t* = 2.747, *p* = 0.014). This hyperlocomotion was reversed in stressed animals treated with 10 mg/kg of Suvorexant (Holm–Sidak: *t* = 2.555, *p* = 0.014; *n* = 13 rats). Taken together, these data suggest two-day inescapable foot-shock stress can exacerbate MK-801 induced hyperlocomotion and that this aberrant behavior can be reversed with Suvorexant.

### Selective OX_1_R and OX_2_R antagonists both reverse aberrant VTA dopamine neuron population activity following stress

**Fig. 3:**
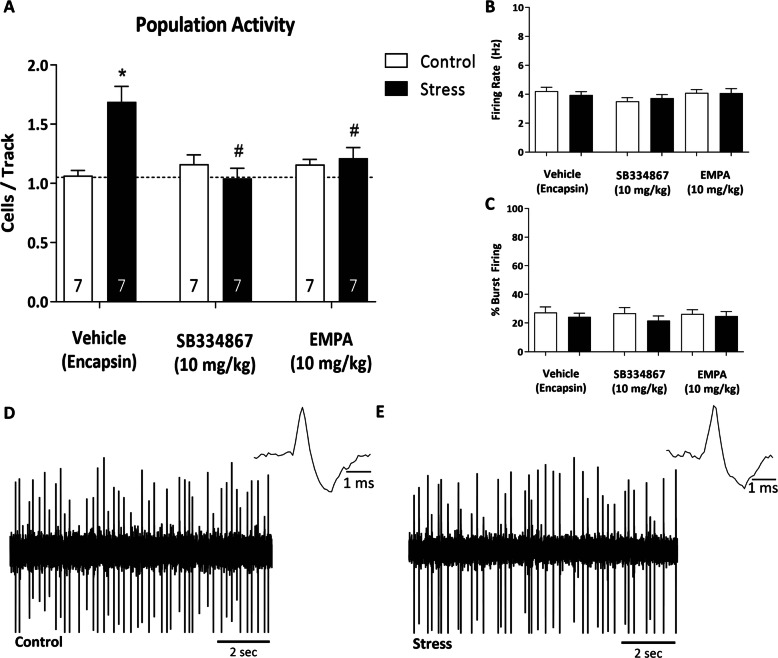
Selective OX_1_R and OX_2_R antagonists reverse stress-induced aberrant VTA dopamine neuron activity. In vivo extracellular electrophysiology was used to record dopamine cell activity in the VTA, following stress. **a** Two-day inescapable foot-shock resulted in a significant increase in the number of spontaneously active dopamine neurons per electrode track. This increase was reversed by both SB334867 and EMPA. *p < 0.05 compared with control/vehicle; #p < 0.05 compared with stress/vehicle. There were no significant differences in **b** firing rate or **c** bursting pattern of recorded dopamine cells. Representative traces from control animals (**d**) and two-day inescapable shock animals (**e**). *n* = 7 rats per group.

After observing that blockade of both OX_1_R and OX_2_R, with the DORA Suvorexant, reversed stress-induced deficits in both dopamine neuron population activity and dopamine-dependent behaviors, we investigated the ability of each receptor subtype to reverse behavioral and physiological changes after stress. Similar to our Suvorexant experiments, we found a main effect of stress (Fig. 3; two-way ANOVA; factors: stress × drug; *F*_(1, 41)_ = 7.130; *t* = 2.670; *p* = 0.011, *n* = 7 rats/group). Stress/vehicle animals displayed an increase in the number of spontaneously active dopamine cells per track compared to control/vehicle animals (Holm–Sidak: *t* = 5.224, *p* = <0.001; control/vehicle: 1.06 ± 0.04 cells per track; stress/vehicle; 1.69 ± 0.13 cells per track). Interestingly, this effect was attenuated following administration of either an OX_1_R antagonist, SB334867 (10 mg/kg), or an OX_2_R antagonist, EMPA (10 mg/kg). SB334867/stress animals displayed a complete reversal of aberrant dopamine neuron activity compared to stress/vehicle animals (*p* = <0.001; 1.04 ± 0.09 cells per track), as did EMPA/stress animals (*p* = <0.001; 1.21 ± 0.09 cells per track). Additionally, we examined two other parameters of dopamine cell activity; firing rate and percentage bursting. There were no significant differences in firing rate (control/vehicle: *n* = 50 neurons; 4.22 ± 0.26 Hz; stress/vehicle: *n* = 89 neurons; 3.95 ± 0.23 Hz; control/SB334867: n = 53 neurons; 3.51 ± 0.25 Hz; stress/SB334867: *n* = 49 neurons; 3.72 ± 0.25 Hz; control/EMPA: *n* = 63 neurons; 4.09 ± 0.23 Hz; stress/EMPA: *n* = 57 neurons; 4.07 ± 0.31 Hz) or percentage bursting (control/vehicle: *n* = 50 neurons; 27.35 ± 3.78%; stress/vehicle: *n* = 89 neurons; 24.31 ± 2.58%; control/SB334867: *n* = 53 neurons; 26.85 ± 3.89%; stress/SB334867: *n* = 49 neurons; 21.59 ± 3.34%; control/EMPA: *n* = 63 neurons; 26.28 ± 2.97%; stress/EMPA: *n* = 57 neurons; 24.78 ± 3.24%). Collectively, these data suggest blockade of either OX_1_R or OX_2_R can alleviate aberrant dopamine cell activity, following stress. However, given the sedative effects of OX_2_R antagonists, selective OX_1_R antagonists may be a more feasible treatment option for PTSD and comorbid psychosis.

### OX_1_R antagonist, but not OX_2_R antagonist, reverses stress-induced deficits in PPI

**Fig. 4:**
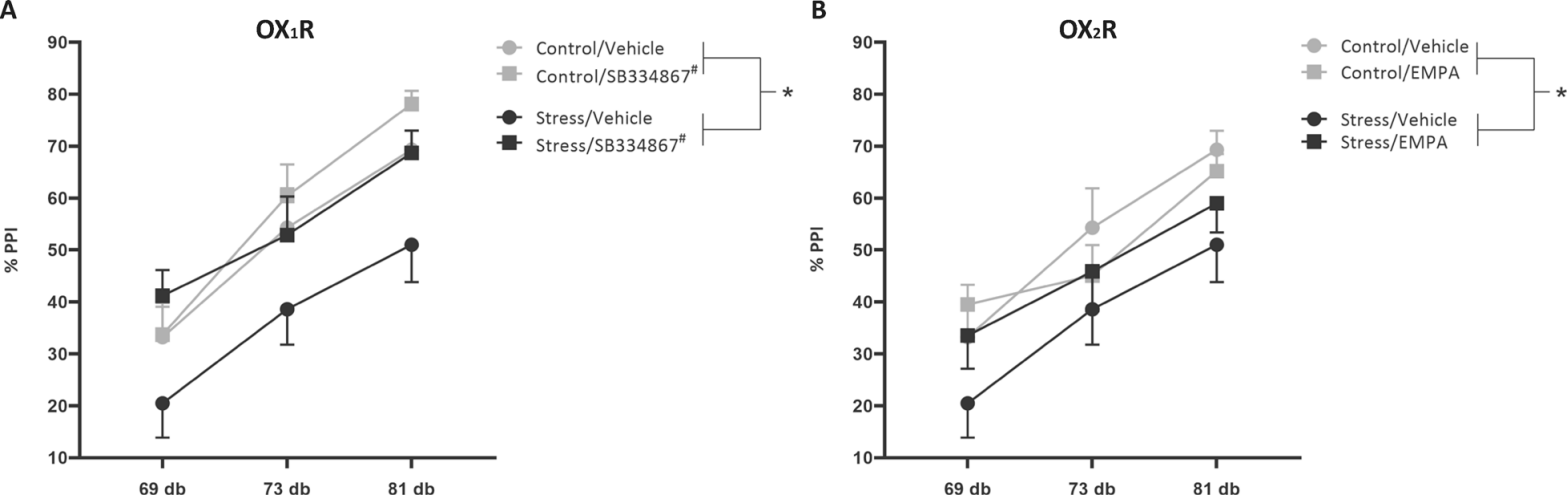
OX_1_R antagonist, not OX_2_R antagonist, alleviates stress-induced deficits in PPI. As previously observed, two-day inescapable foot-shock produces deficits in PPI. These deficits were attenuated following systemic administration of the OX_1_R antagonist, SB334867 (**a**) but not following systemic administration of the OX_2_R antagonist, EMPA (**b**). *p < 0.05 denotes stress animals were significantly different from control animals; ^#^p < 0.05 compared with vehicle-treated animals.

After demonstrating the ability of both OX_1_R and OX_2_R receptor antagonists to alleviate stress-induced increases in VTA dopamine neuron activity, we examined the ability of the OX_1_R antagonist, SB334867, and the OX_2_R antagonist, EMPA to reverse stress-induced deficits in PPI. Unlike our observations from our in vivo dopamine electrophysiology experiments, we found that only SB334867 was able to reverse deficits in PPI (Fig. 4). As expected, we observed a main effect of pre-pulse intensity (Fig. 4; *p* < 0.001; *n* = 10 rats/group). Similar to previous observations, we found a main effect of stress (Fig. 4; three-way ANOVA; factors: stress × drug × dB; *F*_(1,179)_ = 7.385; *t* = 2.717; *p* = 0.007). Additionally, we observed a main effect of drug, in which SB334867 treated animals displayed a statistically significant difference in PPI than stress/vehicle animals (*t* = 3.358, *p* = 0.003, *n* = 10 animals/group) but EMPA treated animals did not differ statistically from stress/vehicle-treated animals (although a trend was observed). No differences were observed in average startle response in animals treated with SB334867 (Holm–Sidak: *t* = 0.149, *p* = 0.968) or EMPA (*t* = 0.228, *p* = 0.994) compared to vehicle-treated animals. These data further support the role of OX_1_R antagonists in alleviating symptoms of psychosis in PTSD patients.

## Discussion

In the current experiments, we utilized the FDA-approved DORA, Suvorexant (trade name: Belsomra)^45^, a selective OX_1_R antagonist, SB334867, and a selective OX_2_R antagonist, EMPA, to demonstrate that stress-related physiological and behavioral alterations observed in a stress-induced model can be reversed by systemic orexin receptor blockade. In order to model PTSD-like pathophysiology in a rodent, we used the two-day inescapable foot-shock procedure, which has been shown to produce robust behavioral and physiological changes, such as avoidance and anxiety-like behavior, hyperarousal and aggression, and alterations in sleep^28–30^. Here we demonstrate that two-day, inescapable foot-shock produces specific increases in VTA dopamine population activity as well as deficits in dopamine-dependent behaviors (PPI and stimulant-induced hyperlocomotion). Furthermore, systemic administration of either the DORA, Suvorexant, or the selective OX_1_R antagonist, SB334867, completely alleviated stress-induced physiological and behavioral changes. Our results suggest that targeting the orexin system may be a novel therapeutic approach for the treatment of PTSD and comorbid psychosis.

Although the presentation of PTSD and comorbid psychosis is highly heterogeneous, there are multiple brain regions consistently implicated in the disorder, including the thalamus^27,46–49^. The thalamus is composed of multiple nuclei, but the afferent and efferent projections of the PVT position this particular thalamic nucleus to regulate symptoms associated with PTSD and comorbid psychosis. Activation of the PVT has been shown to occur following numerous stressful and aversive events, such as restraint, exposure to predator scent, and foot-shock^50–52^. Additionally, both structural and metabolic changes within the thalamus are observed in patients with psychotic symptoms^53^. Our laboratory has recently demonstrated that increased glutamatergic transmission from the PVT, specifically, to the NAc causes an increase in the number of spontaneously active dopaminergic neurons in the VTA, through a multisynaptic pathway^12^. Symptoms of psychosis consistently correlate with an increase in mesolimbic dopamine transmission, which can be observed in both humans, using PET imaging^9^, as well as in animal models of psychosis, using in vivo electrophysiology^10^. While it is difficult to model and measure symptoms of psychosis in a rodent, such as hallucinations and delusions, it is possible to measure changes in dopamine neuron activity. Using in vivo extracellular electrophysiology, we are able to evaluate aspects of dopamine cell activity (population activity, firing rate and burst firing)^11^. We have previously demonstrated, in a rodent model used to study psychosis, that intracranial administration of the DORA, TCS1102, into the PVT can restore dopamine system function, suggesting the PVT may be a novel therapeutic target for the treatment of psychosis^27^. Taken together, data from previous studies suggest the PVT becomes activated following stressful events^50–52^ and that this aberrant activation may contribute to increased dopamine neuron population activity within the VTA that ultimately underlies symptoms of psychosis.

Orexin-containing neurons originate in the lateral hypothalamus and innervate a variety of brain regions, including the PVT^14^. These neurons release orexin neuropeptides, OXA and OXB, which bind to and activate two Gαq-protein-coupled receptors, OX_1_R and OX_2_R^16^, which, once activated, increase intracellular calcium concentrations^54,55^. Although orexins were originally discovered for their role in regulating sleep and wakefulness^14,22^, they have now been implicated in host of other behaviors, including avoidance behavior^56^, drug-seeking behavior^23,24^, and psychosis^25^. Of interest to the current project, is the ability of both OXA and OXB to dose-dependently modulate PVT neuron activity and the ability of a DORA to reverse aberrant dopamine neuron activity when administered directly in the PVT^26,27^. Thus, we posit that pharmacological blockade of orexin receptors may inhibit stress-induced activation of the PVT and subsequently restore downstream dopamine system function and associated behaviors in a rodent model of PTSD and comorbid psychosis.

Indeed, we found that systemic administration of the FDA-approved DORA, Suvorexant, was able to reverse both aberrant dopamine neuron population activity and behavioral deficits in PPI and stimulant-induced locomotor activity, following stress. It is important to note that although Suvorexant is currently prescribed as a hypnotic, it does not significantly alter the time taken to fall asleep^57^. Rather, Suvorexant administration provides relief for insomnia by decreasing the time to wake after sleep onset and increasing the total time spent asleep^58^. Our results suggest lower, non-sedative doses of Suvorexant are sufficient to alleviate behavioral correlates of psychosis. Indeed, in our study, the administration of Suvorexant (10 mg/kg) did not significantly reduce baseline locomotor activity (distance traveled), as shown in Fig. 2D, suggesting that this dose does not produce overt sedative effects. Conversely, initial pilot studies demonstrated that the high dose of Suvorexant (30 mg/kg) dramatically reduced spontaneous locomotor activity (by >80%), prompting us to halt any further behavioral assays using this dose. This is consistent with the literature, in which 30 mg/kg of Suvorexant has been reported to reduce baseline locomotor activity^59^. Although 30 mg/kg of Suvorexant resulted in behavioral sedation in our pilot experiment, we did not observe decreases in dopamine neuron population activity in our control group, which could be expected in sedation alone resulted in decreased dopamine neuron activity. Taken together, these data suggest lower, non-sedating doses of Suvorexant may be effective at treating comorbid psychosis.

After demonstrating that antagonizing both OX_1_R and OX_2_R with the DORA, Suvorexant, restores stress-induced increases in VTA dopamine neuron activity, we examined the ability of each orexin receptor subtype to selectively reverse aberrant dopamine neuron activity. Although both orexin receptors play a role in the sleep-wake cycle, OX_2_R is necessary for wakefulness, while OX_1_R plays a less critical role^60^. Evidence from both human and animal studies have shown that antagonism or loss of OX_2_R, but not OX_1_R, results in narcoleptic symptoms^61–63^. Thus, selective antagonism of OX_1_R in the treatment of PTSD and comorbid psychosis would be ideal to avoid sedation while still decreasing aberrant dopamine transmission. We therefore examined the role of each orexin receptor in modulating aberrant dopamine transmission following stress. Given that both orexin receptors are expressed in the PVT and that direct injections of OXA and OXB into the PVT can cause significant increases in dopamine neuron population activity^26^, we posited that selective antagonism of either receptor would reverse stress-induced increases in VTA dopamine neuron activity. After administration of the selective OX_1_R antagonist, SB334867 (10 mg/kg)^64^, and selective OX_2_R antagonist, EMPA (10 mg/kg)^36^, we found that both compounds were able to reverse aberrant dopamine system function in our rodent model of PTSD and psychosis. These studies provide further evidence that targeting the orexin receptors in the PVT is useful at treating dopamine dysfunction in a rodent model of PTSD and comorbid psychosis.

Lastly, we evaluated the ability of the selective orexin receptor compounds to reverse a dopamine-dependent behavior associated with psychosis. PPI is a commonly used assay to assess psychosis in humans and that can be effectively measured in rats^32^. Although both SB334867 (OX_1_R) and EMPA (OX_2_R) were able to reverse aberrant dopamine neuron population activity, only SB334867 was able to reverse stress-induced deficits in PPI (although a trend was observed with EMPA). While sensorimotor gating is dependent on dopamine release in the NAc, the ability to regulate PPI is not exclusive to this brain region. Brain regions such as the hippocampus, mPFC, and amygdala have also been shown to contribute to deficits in PPI^32^ and previous studies have demonstrated orexin receptor expression and orexin receptor signaling in these regions is altered following stress^65–67^. Furthermore, the expression of OX_1_R and OX_2_R differs within these regions. Namely, OX_1_R is highly expressed in CA1 and CA2 hippocampal fields, mPFC subregions (ie. the infralimbic cortex), and various amygdala nuclei while OX_2_R shows minimal expression in all these regions^18^, suggesting antagonism of OX_1_R may have direct effects on circuitry responsible for sensorimotor gating. Conversely, dopamine neuron activity in the VTA has been shown to be regulated by the PVT^12,27^, which is densely innervated by orexin containing neurons and displays substantial expression of both OX_1_R and OX_2_R. These differences in expression of orexin receptor subtypes could explain the differential effects of OX_1_R and OX_2_R antagonists on PPI and VTA dopamine neuron activity but further investigation is warranted to fully elucidate this finding.

In conclusion, we have demonstrated that by targeting orexin receptors with the FDA-approved DORA, Suvorexant (30 mg/kg), OX_1_R antagonist, SB334867, and the OX_1_R antagonist, EMPA, we are able to restore normal dopamine system function in a rodent model of stress-induced pathophysiology relevant to PTSD and comorbid psychosis. Further, a low, non-sedating dose of the FDA-approved antagonist, Suvorexant, was able to reverse PPI deficits and eliminate increased sensitivity to psychomotor stimulants observed in the stress model. Additionally, the OX_1_R antagonist, SB334867, can also alleviate deficits in PPI observed in our model. Taken together, these findings suggest that pharmacological blockade of orexin receptors, specifically OX_1_R, may be a novel therapeutic approach for the treatment of PTSD and comorbid psychosis.
